# Lipid balance and chemoresistance in cancer cells

**DOI:** 10.7554/eLife.110231

**Published:** 2026-01-12

**Authors:** Kaori Kanemaru, Yoshikazu Nakamura

**Affiliations:** 1 Department of Applied Biological Science, Faculty of Science and Technology, and the Division of Aging Biology, Research Institute for Science and Technology, Tokyo University of Science Tokyo Japan

**Keywords:** cancer, cholesterol, epithelial-mesenchymal transition, sphingomyelins, snail, chemotherapy, Human, Mouse

## Abstract

A shift in the balance between two lipids – cholesterol and sphingomyelin – makes hybrid epithelial-mesenchymal cancer cells less responsive to certain chemotherapy drugs.

**Related research article** Matsumoto A, Inoko A, Tanaka T, Konishi GI, Hosoda W, Kojima T, Ohnishi K, Ikenouchi J. 2024. Chemotherapy resistance due to epithelial-to-mesenchymal transition is caused by abnormal lipid metabolic balance. *eLife* **13**:RP104374. doi: 10.7554/eLife.104374.

Cells are enclosed by a thin plasma membrane made mostly of lipids. This layer keeps the contents of the cell in place and controls which molecules can enter or leave. Two of the lipids in this membrane, cholesterol and sphingomyelin, often work together (Kraft, 2017; Endapally et al., 2019). However, too much “free” cholesterol can be harmful for a cell, so the level of this lipid is kept under strict control (Wang and Tontonoz, 2018).

The relative levels of cholesterol and sphingomyelin are also important in cancer cells. These cells often escape the harsh conditions found in tumours by switching into different cellular states. In the epithelial-to-mesenchymal transition, for example, epithelial cells lose their cell polarity and ability to adhere to each other to become mesenchymal cells, which are mobile. The epithelial-to-mesenchymal transition is linked to metastasis and poor prognosis, and cells that activate this transition often withstand chemotherapy better than their epithelial counterparts (Fontana et al., 2024). However, the processes by which this transition leads to chemoresistance are not fully understood.

In many tumours, the transition does not always progress to a fully mesenchymal state. Instead, cells stop in a hybrid epithelial–mesenchymal (E/M) state in which they keep some epithelial features while acquiring mesenchymal ones (Liao and Yang, 2020; Rashid et al., 2022). Now, in eLife, Junichi Ikenouchi (Kyushu University) and colleagues – including Atsushi Matsumoto as first author – report the results of experiments on mice and human cell lines which show that hybrid E/M cells can tolerate an otherwise harmful imbalance between cholesterol and sphingomyelin (Matsumoto et al., 2024).

The researchers studied hybrid E/M cells induced by Snail, a transcription factor that regulates the epithelial-to-mesenchymal transition during embryonic development and also in cancer cells (Figure 1). Snail lowers the levels of enzymes that make a form of sphingomyelin that pairs especially well with cholesterol. The plasma membrane of the cell is therefore pushed into a state that is relatively rich in cholesterol and relatively poor in sphingomyelin. This imbalance activates a lipid-sensing receptor in the cell nucleus called LXR (short for liver X receptor), which reduces the level of cholesterol by inducing a transport protein called ABCA1 that exports cholesterol from the cell. In parallel, excess cholesterol is converted into cholesterol esters (a process called esterification) which are then stored in lipid droplets.

**Figure 1.**
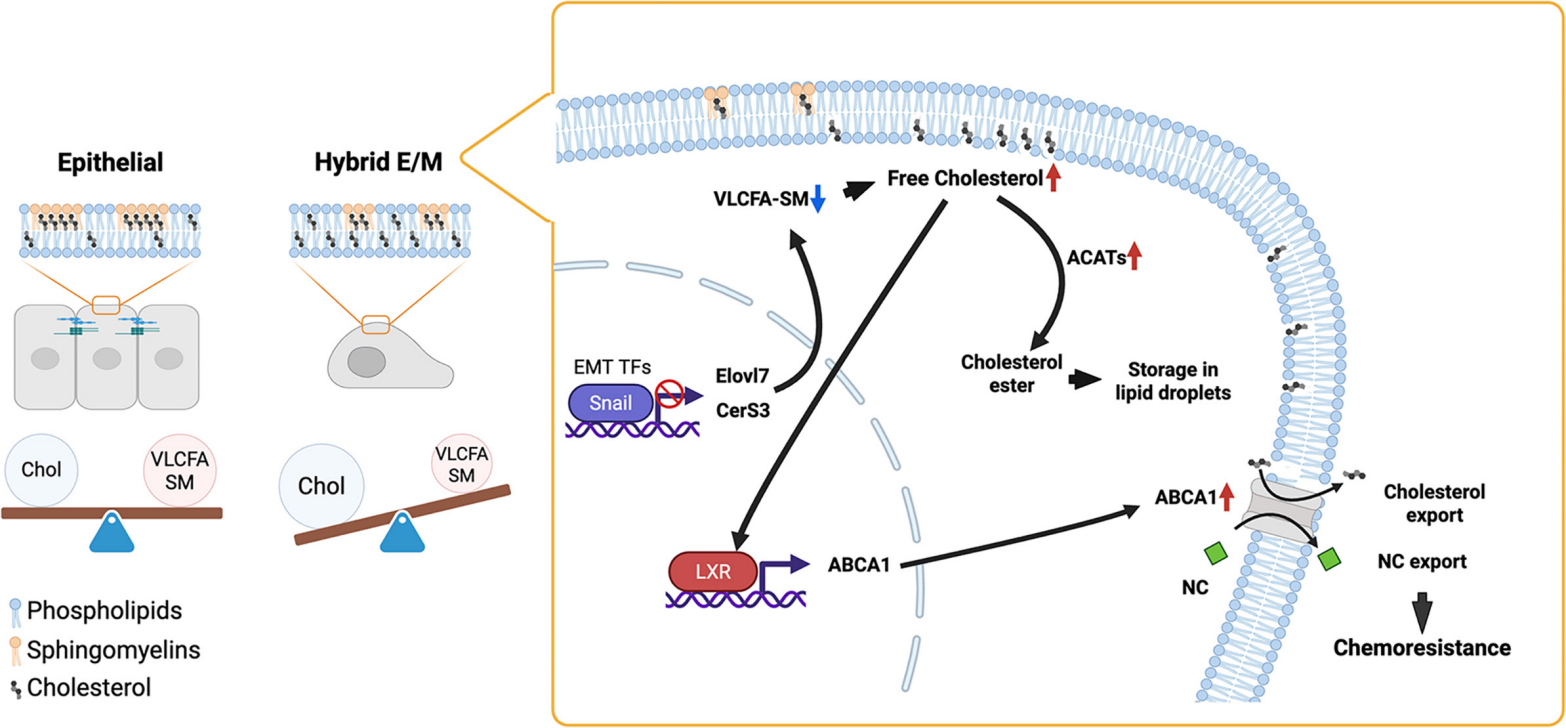
Lipid imbalance in hybrid epithelial–mesenchymal cancer cells. In epithelial cells (left), cholesterol (Chol; black shapes) and very-long-chain fatty acid sphingomyelin (VLCFA-SM; peach) form a balanced partnership within the plasma membrane, whereas cells in a hybrid epithelial–mesenchymal (E/M) state have membranes that are rich in cholesterol and poor in sphingomyelin. Activation of a transcription factor called Snail has two effects (right): i) it induces entry into the hybrid E/M state; ii) it suppresses the enzymes that are responsible for the synthesis of VLCFA-SM (such as Elovl7 and CerS3), which leads to an increase in the level of free cholesterol. The resulting lipid imbalance activates the nuclear receptor LXR, which in turn induces the transport protein ABCA1 to promote cholesterol export from the cell. In parallel, excess cholesterol is esterified by enzymes called ACATs and stored in lipid droplets. At the same time, ABCA1 also promotes the export of the chemotherapy drug nitidine chloride (NC; green), thus making hybrid E/M cells less responsive to chemotherapy. Created with BioRender.com.

Put simply, Snail pushes cells into a state in which cholesterol is too abundant relative to sphingomyelin. Hybrid E/M cells can survive in this state by relying strongly on LXR-driven cholesterol export and on cholesterol esterification. However, when LXR signalling is blocked, or when the enzymes that promote esterification are inhibited, the hybrid E/M cells are harmed much more than control cells, both in cell cultures and in mice with kidney tumours.

This weakness appears to be specific to the hybrid E/M state rather than to mesenchymal cells in general. In the models examined by Matsumoto et al., fully mesenchymal cells appear to cope with extra cholesterol by increasing sphingomyelin synthesis and keeping the cholesterol–sphingomyelin ratio within a narrow range, so they don’t have to depend on cholesterol export or esterification. Snail-induced hybrid E/M cells, in contrast, seem unable to make more sphingomyelin, so they have to compensate by constantly pumping cholesterol out of the cell and/or storing it in lipid droplets. This reveals a particular metabolic vulnerability of the hybrid E/M state: if the export and/or storage process is disturbed, the cells are unable to keep the level of free cholesterol under control.

How does this connect to chemotherapy? ABCA1, the protein that exports cholesterol from hybrid E/M cells, can also export nitidine chloride (Iwasaki et al., 2010), the chemotherapy drug used by Matsumoto et al. in their study. Snail-expressing hybrid E/M cells are more resistant to nitidine chloride than parental epithelial cells, but blocking LXR signalling or inhibiting ABC transporters restores the sensitivity of hybrid E/M cells to this drug. Therefore, the main reason that E/M cells export and/or store cholesterol is to cope with the lipid imbalance created by Snail. However, once they are active, these pathways also export drugs that ABCA1 can bind to, such as nitidine chloride.

In this view, chemoresistance is not the main purpose of the epithelial-to-mesenchymal transition, but a side effect of how hybrid E/M cells adapt to cholesterol stress. Beyond the specific molecules involved, this study carries a broader message. Rather than focusing only on the total amount of a given lipid, it highlights the importance of lipid balance – here, the ratio between cholesterol and sphingomyelin – as a key factor that shapes cell behaviour. It also illustrates how a change in cell state can create new metabolic weak points.

The work by Matsumoto et al. therefore adds a new layer to our understanding of the epithelial-to-mesenchymal transition. When Snail is activated in an epithelial cell, the consequences extend beyond changes in cell shape and a reduction in the ability to adhere to other cells: the partnership between cholesterol and sphingomyelin is disturbed, and the cells become more dependent on pathways that export and store cholesterol. Targeting this dependency – for example, by combining chemotherapy with drugs that interfere with cholesterol handling – could provide a way to exploit the very metabolic adaptation that helps hybrid E/M cells endure.
